# Molecular docking analysis of piperine with CDK2,CDK4,Cyclin D and Cyclin T proteins

**DOI:** 10.6026/97320630016359

**Published:** 2020-05-31

**Authors:** Umapathy Vidhya Rekha, M Anita, Govindaraj Jayamathi, K Sadhana, Subramanian Deepa, Sajid Hussain, J Bhuvaneswari, V Ramya, Jayaraman Selvaraj, NS Naveenraj

**Affiliations:** 1 Department of Public Health Dentistry, Sree Balaji Dental College and Hospital, Pallikaranai, Chennai-600 100, India; 2 Department of Biochemistry, Sree Balaji Dental College and Hospital, Pallikaranai, Chennai-600 100, India; 3 Department of Periodontics, Sree Balaji Dental College and Hospital, Pallikaranai, Chennai-600 100, India; 4 Department of Biochemistry, Saveetha Dental College and Hospitals, Saveetha Institute of Medical and Technical Sciences, Saveetha University, Chennai - 600 077, India; 5 Department of Public Health Dentistry, Ragas Dental College and Hospital, Chennai India

**Keywords:** Piperine, CDK2, CDK4, Cyclin D and Cyclin T, cell cycle regulators, oral cancer, molecular docking

## Abstract

Piperine is a component of Piper nigrum (Black pepper). It is well known in ayurvedic formulations. Piperine is a bioenhancer as it reduces the activity of drug-metabolizing enzymes in rodents and thereby enhancing the plasma concentrations of several drugs, including the Pglycoprotein substrates. Therefore, it is of interest to understand the molecular docking interactions of piperine with several cell cycle proteins such as Cyclin dependent kinase 2 (CDK2), Cyclin-dependent kinase 4 (CDK4), Cyclin D and Cyclin T for further consideration in drug discovery related to oral cancer.

## Background

Oral cancer is described as the cancer of lips, tongue, cheeks, floor of the mouth, hard and soft palate, sinuses and pharynx (throat) and it is life threatening if not identified and treated [1].Oral squamous cell carcinoma is a clinical diagnostic challenge to the dental practitioner, during the early stage of development.Such cancers are linked with smoking and alcohol abuse [2]. A 2 to 3-fold death rate increases have been documented in eastern and central European countries in the past 3 decades [3]. In India, oral cancer, ranks first among males and is the third most frequent one among females in several areas [4]. Oral cancer is the 6th mainly frequent cancer for both sexes in the universal population, and the third most frequent cancer in developing nations [5].

Regulation of the cell cycle involving cell-signaling pathways is linked with tumor targets for drug discovery. Thus, cell cycle phases give promise for the development of drug like molecules for cancer treatment. Cell cycle progression five phases namely G0 (gap 0), G1 (gap 1), S (DNA synthesis), G2 (gap 2), and M (mitosis). Two important checkpoints are at the G1/S and G2/M limits [6]. Known anti-cancer drugs are DNA damaging agents resulting in chemo resistance. Thus, design and development of anti-cancer drugs is gaining momentum in recent years [7]. Screening of natural compounds for drug discovery to combat several forms of cancer is common in modern medical research and development. Piper nigrum, generally known as black pepper is utilized as a health related remedy and is considered as King of spices [8]. Therefore, it is of interest to document the molecular docking analysis data of piperine with the cell cycle proteins such as CDK2, CDK4, Cyclin D and Cyclin T to combat oral cancer.

## Materials and Methods

### Protein preparation:

The structures of the cell cycle regulatory proteins such as CDK2 (PDB ID: 1W98), CDK4 (PDB: 3G33), Cyclin D (PDB ID: 2W9F) Cyclin T (PDB ID: 3BLR) were downloaded from PDB [9]. The data was processed by removing the hetero-atoms and water molecules for docking using the PATCH DOCK server.

### Ligand preparation:

The piperine 3D was downloaded from pubchem database in SDF format and it was transformed to PDB file format using the Online Smile Translator. Energy minimizations of ligands were completed using the ChemBio 3D Ultra 12.0 software.

### Molecular docking:

PatchDock is a geometry oriented molecular docking algorithm for docking scores by identifying and scoring interacting amnio acids and atomic contact energy (ACE) for the given ligands [10,11]. The server returns data using e-mail. The top scoring interaction was further analyzed using Ligplot.

## Results and discussion:

**Figure 1:**
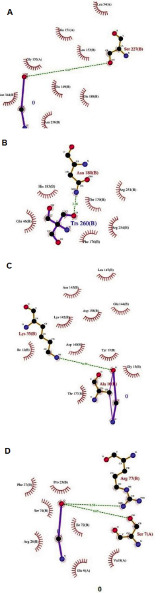
Interaction of piperine with (A) CDK2, (B) CDK4, (C) Cyclin D and (D) Cyclin T proteins shown using Ligplot

Data from the molecular docking analysis of piperine with the cell cycle proteins such as CDK2, CDK4, Cyclin D and Cyclin T using the PatchDock server is given in (Table 1) using models described elsewhere [12-13]. Several quantifiable interaction features between piperine and the target proteins is documented. Data suggest optimal binding of piperine with the cell cycle proteins analysed. The atomic interaction between piperine with the cell cycle proteins such as CDK2, CDK4, Cyclin D and Cyclin T is developed using ligplot as shown in (Figure 1).(Figure 1) illustrates the optimal binding features with nice hydrogen bonds between ligand piperine and the protein targets for further in vivo and in vitro consideration.

## Conclusions:

We document the molecular docking analysis data of piperine with the cell cycle proteins such as CDK2, CDK4, Cyclin D and Cyclin T for further consideration to combat oral cancer.

## Figures and Tables

**Table 1:** Molecular docking results obtained through Patch dock server

S. No	Protein name	Score (kca l/mol)	Energy	Interacting amino acids residues	H bond length	No of non-bonded interaction
1	CDK 2	5190	-120.65	SER 227 OG.-O	3.19	60
2	CDK 4	4708	-96.49	ASN 180 NH-0	3.26	23
3	Cyclin D	4496	-76.17	LYS 35 NZ-O	2.62	98
4	Cyclin T	4326	-111.42	SER 7 OG-O	1.5	139
				ARG 77 NH-O	2.08	

## References

[1] Elango JK (2006). Asian Pac J Cancer Prev..

[2] Julien JA (1995). Community Dent Health..

[3] Coleman MP (1993). IARC Sci Publ.

[4] Macfarlane GJ (1994). Cancer Causes Control.

[5] La Vecchia C (1997). Oral Oncol..

[6] Dominguez-Brauer C (2015). Mol Cell..

[7] Cicenas J (2014). Cancers (Basel)..

[8] Yim H (2013). Anticancer Drugs..

[9] Bernstein FC (1978). Arch Biochem Biophys.

[10] Schneidman-Duhovny D (2003). Proteins..

[11] Schneidman-Duhovny D (2005). Nucleic Acids Res..

[12] Vijayalakshmi P (2014). Interdiscip Sci..

[13] Yuriev E (2013). J Mol Recognit..

